# *catena*-Poly[[[(2-phenyl­acetato-κ*O*)zinc(II)]bis­[μ-4,4′-(disulfanedi­yl)dipyridine-κ^2^ *N*:*N*′]] monohydrate]

**DOI:** 10.1107/S1600536810021331

**Published:** 2010-06-16

**Authors:** Jie Zhang, Wei Xu

**Affiliations:** a State Key Laboratory Base of Novel Functional Materials and Preparation Science, Center of Applied Solid State Chemistry Research, Ningbo University, Ningbo, Zhejiang 315211, People’s Republic of China

## Abstract

In the title compound, {[Zn(C_8_H_7_O_2_)_2_(C_10_H_8_N_2_S_2_)_2_]·H_2_O}_*n*_, the Zn^II^ atom is coordinated by four N atoms from four 4,4′-(disulfanedi­yl)dipyridine (bpds) ligands and two O atoms from two 2-phenyl­acetate anions in a distorted octa­hedral coordination geometry. The two bpds ligands of the same axial chirality bridge Zn^II^ atoms, generating repeated rhomboidal chains, which are linked by O—H⋯O hydrogen bonds into a ladder structure.

## Related literature

For coordination chemistry based on pyridyl donor ligands, see: Biradha *et al.* (2006 ▶); Liu *et al.* (2008 ▶); Hernández-Ahuactzi *et al.* (2008 ▶); Ma, Wang, Wang *et al.* (2009 ▶). For bpds compounds, see: Horikoshi & Mochida (2006 ▶); Carballo *et al.* (2008 ▶); Ma, Wang, Hu *et al.* (2009 ▶); Horikoshi & Mikuriya (2005 ▶). For compounds containing phenyl­acetic acid, see: Johnston *et al.* (2008 ▶).
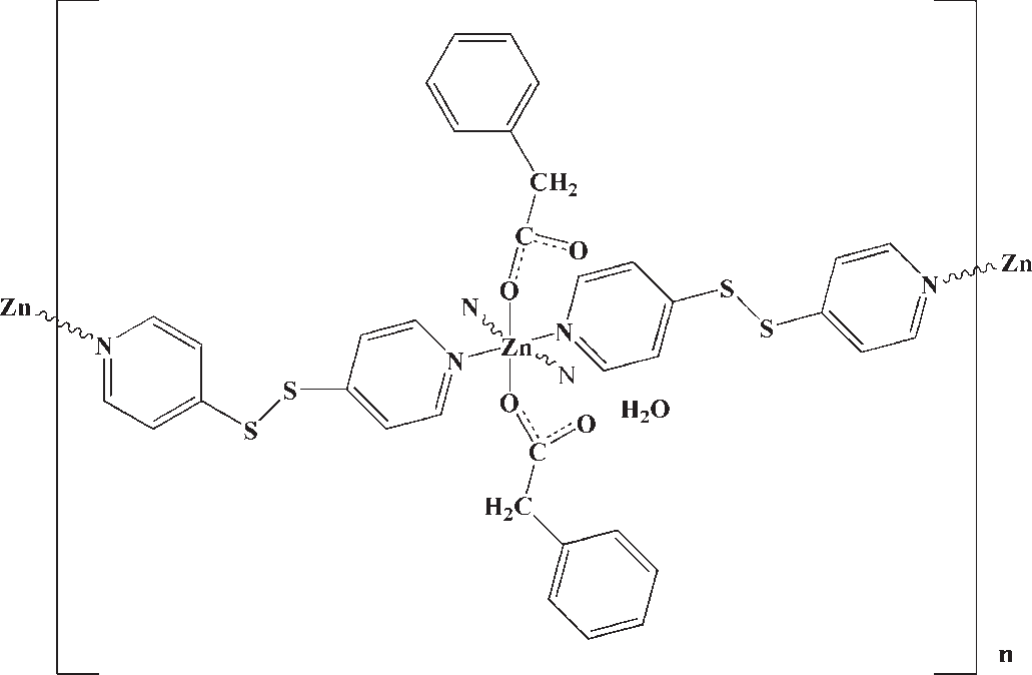


## Experimental

### 

#### Crystal data

[Zn(C_8_H_7_O_2_)_2_(C_10_H_8_N_2_S_2_)_2_]·H_2_O
*M* *_r_* = 794.27
Triclinic, 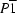

*a* = 9.851 (2) Å
*b* = 11.130 (2) Å
*c* = 18.319 (4) Å
α = 90.38 (3)°
β = 98.88 (3)°
γ = 115.89 (3)°
*V* = 1779.0 (6) Å^3^
*Z* = 2
Mo *K*α radiation
μ = 0.97 mm^−1^
*T* = 295 K
0.51 × 0.41 × 0.36 mm

#### Data collection

Rigaku R-AXIS RAPID diffractometer
Absorption correction: multi-scan (*ABSCOR*; Higashi, 1995 ▶) *T*_min_ = 0.623, *T*_max_ = 0.701
16701 measured reflections
7859 independent reflections
6036 reflections with *I* > 2σ(*I*)
*R*_int_ = 0.038

#### Refinement

*R*[*F* ^2^ > 2σ(*F* ^2^)] = 0.035
*wR*(*F* ^2^) = 0.092
*S* = 1.07
7859 reflections
451 parameters
H-atom parameters constrained
Δρ_max_ = 0.43 e Å^−3^
Δρ_min_ = −0.34 e Å^−3^

### 

Data collection: *RAPID-AUTO* (Rigaku, 1998 ▶); cell refinement: *RAPID-AUTO*; data reduction: *CrystalStructure* (Rigaku/MSC, 2004 ▶); program(s) used to solve structure: *SHELXS97* (Sheldrick, 2008 ▶); program(s) used to refine structure: *SHELXL97* (Sheldrick, 2008 ▶); molecular graphics: *SHELXTL* (Sheldrick, 2008 ▶); software used to prepare material for publication: *SHELXL97*.

## Supplementary Material

Crystal structure: contains datablocks global, I. DOI: 10.1107/S1600536810021331/er2078sup1.cif

Structure factors: contains datablocks I. DOI: 10.1107/S1600536810021331/er2078Isup2.hkl

Additional supplementary materials:  crystallographic information; 3D view; checkCIF report

## Figures and Tables

**Table 1:** Hydrogen-bond geometry (Å, °)

*D*—H⋯*A*	*D*—H	H⋯*A*	*D*⋯*A*	*D*—H⋯*A*
O5—H5c⋯O4^i^	0.81	2.20	2.970 (3)	158
O5—H5d⋯O4	0.80	2.15	2.945 (3)	169

Symmetry code: (i) 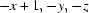.

**Fig. 1.**
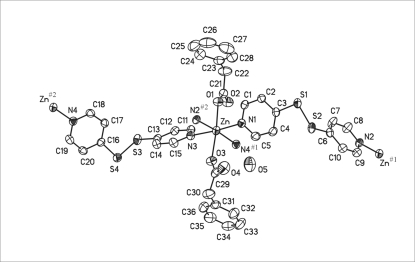
View of the molecular of the title compound, Displacement ellipsoids are drawn at the 45% probability level.

**Fig. 2.**
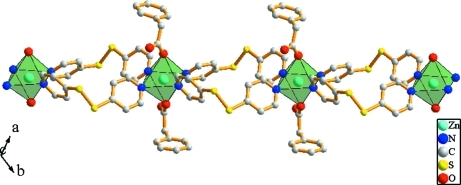
1D repeated rhomboidal chains in the title compound.

**Fig. 3.**
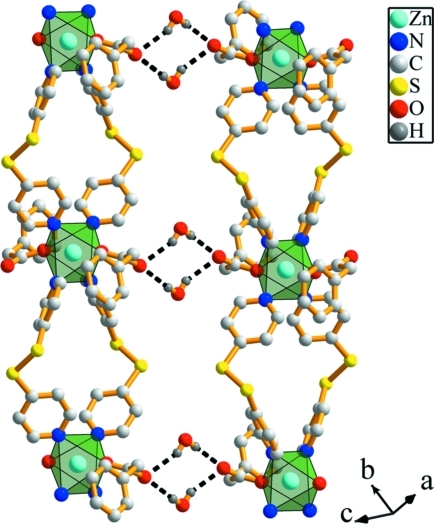
The ladder structure of the title compound.

## supplementary crystallographic information

### Comment

Recently, a variety of pyridyl-donor ligands have been widely employ to construct coordination polymers with intriguing topologies and unexpected properties (Biradha *et al.*, 2006; Liu *et al.*, 2008; Hernández-Ahuactzi *et al.*, 2008; Ma, Wang, Hu *et al.*, 2009). 4,4'-dipyridyl disulfide (bpds) is a bipyridyl-type ligand with a twisted structuret. Addtionally, bpds ligand has axial chirality. A number of coordination polymers containing bpds ligand have been reported (Horikoshi *et al.*, 2006; Carballo *et al.*, 2008; Ma, Wang, Wang *et al.*, 2009). Phenylacetic acid is one of the most common carboxylate ligands and can adopt different coordination modes (Johnston *et al.*, 2008). However, the coordination polymers based on mixed bpds and phenylacetate anion have not been reported to date. In this paper, we report the title Znic polymeric compound, [{Zn(bpds)_2_(C_6_H_5_CH_2_COO)_2_}.H_2_O]_n_ with a 1D repeated rhomboidal chain structure. The unsymmetrical unit of the title compound consisits of one Zn^2+^ cation, two bpds molecules of the same chirality, two phenylacetate anions and one lattice water molecule (Fig.1).The *M-* and *P-* bpds molecules act as bis-monodentate bridging ligands with the C—S—S—C torsion angle being 97.10 (1)° and 93.40 (1)°, respectively, and the corresponding py ring planes form dihedral angles of 89.06 (6)° and 79.42 (6)°. Both crystallographically distinct phenylacetate anions monodentately coordinate to the metal atoms.The Zn atom has a distorted octahedral environment, being surrounded by two nitrogen atoms from two *M-*bpds and two nitrogen atoms from two *P-*bpds in the equatorial plane, and by two oxygen atoms from two crystallographically distinct phenylacetate anions occupying the axial positions. The corresponding bond distances range from 2.111 (2)Å to 2.202 (2) Å, and the bond angles in the region 84.12 (7)—175.94 (7)° deviate from the values of 90° and 180° for an ideal octahedron (table 1). Along the [110] direction, the two bpds ligands of the same chirality bridge Zn atoms to form 1D repeated rhomboidal chains(Fig. 2), which is similar with the structures of the reported zinc coordination polymers based on bpds ligand (Horikoshi *et al.*, 2005). The Zn···Zn separation through bpds ligands is 11.187 Å. The lattice water form hydrogen bonds to the uncoordinated carboxylate oxygen atoms of two different phenylacetate anions. In this way, the adjacent chains are linked by water molecules to give a ladder structure.

### Experimental

0.0750 g (0.25 mmol) Zn(NO_3_)_2_.6H_2_O and 0.0345 g (0.25 mmol) phenylacetic acid were succesively dissolved in a stirred aqueous ethanolic solution consisting of 5 ml EtOH and 10 ml H_2_O, to which 0.5 ml 1.0 M NaOH was added. The formed white suspension was stirred at 80°C for 30 min and then added was an ethanolic solution of 0.0570 g (0.25 mmol) 4,4'-dipyridyldisulfide in 5 ml EtOH. The final mixture was further stirred at 75°C for 1 h and filtered off. The colorless filtrate (pH=6.02) was left standing at room temperature for one week affording colorless block-like crystals (yield: 4 mg).

### Refinement

H atoms bonded to C atoms were palced in geometrically calculated position and were refined using a riding model, with *U*_iso_(H) = 1.2 Ueq(C). H atoms attached to O atoms were found in a difference Fourier synthesis and were refined using a riding model, with the O—H distances fixed as initially found and with *U*_iso_(H) values set at 1.2 *U*eq(O).

### Crystal data

**Table:**

[Zn(C_8_H_7_O_2_)_2_(C_10_H_8_N_2_S_2_)_2_]·H_2_O	*Z* = 2
*M**_r_* = 794.27	*F*(000) = 820
Triclinic, *P*1	*D*_x_ = 1.483 Mg m^−^^3^
Hall symbol: -P 1	Mo *K*α radiation, λ = 0.71073 Å
*a* = 9.851 (2) Å	Cell parameters from 16701 reflections
*b* = 11.130 (2) Å	θ = 3.1–27.5°
*c* = 18.319 (4) Å	µ = 0.97 mm^−^^1^
α = 90.38 (3)°	*T* = 295 K
β = 98.88 (3)°	Block, colorless
γ = 115.89 (3)°	0.51 × 0.41 × 0.36 mm
*V* = 1779.0 (6) Å^3^	

### Data collection

**Table:**

Rigaku R-AXIS RAPID diffractometer	7859 independent reflections
Radiation source: fine-focus sealed tube	6036 reflections with *I* > 2σ(*I*)
graphite	*R*_int_ = 0.038
Detector resolution: 0 pixels mm^-1^	θ_max_ = 27.5°, θ_min_ = 3.1°
ω scans	*h* = −12→12
Absorption correction: multi-scan (*ABSCOR*; Higashi, 1995)	*k* = −11→14
*T*_min_ = 0.623, *T*_max_ = 0.701	*l* = −23→23
16701 measured reflections	

### Refinement

**Table:**

Refinement on *F*^2^	Primary atom site location: structure-invariant direct methods
Least-squares matrix: full	Secondary atom site location: difference Fourier map
*R*[*F*^2^ > 2σ(*F*^2^)] = 0.035	Hydrogen site location: inferred from neighbouring sites
*wR*(*F*^2^) = 0.092	H-atom parameters constrained
*S* = 1.07	*w* = 1/[σ^2^(*F*_o_^2^) + (0.0395*P*)^2^ + 0.3693*P*] where *P* = (*F*_o_^2^ + 2*F*_c_^2^)/3
7859 reflections	(Δ/σ)_max_ = 0.001
451 parameters	Δρ_max_ = 0.43 e Å^−^^3^
0 restraints	Δρ_min_ = −0.34 e Å^−^^3^

### Special details

**Table:**

Geometry. All esds (except the esd in the dihedral angle between two l.s. planes) are estimated using the full covariance matrix. The cell esds are taken into account individually in the estimation of esds in distances, angles and torsion angles; correlations between esds in cell parameters are only used when they are defined by crystal symmetry. An approximate (isotropic) treatment of cell esds is used for estimating esds involving l.s. planes.
Refinement. Refinement of *F*^2^ against ALL reflections. The weighted *R*-factor wR and goodness of fit *S* are based on *F*^2^, conventional *R*-factors *R* are based on *F*, with *F* set to zero for negative *F*^2^. The threshold expression of *F*^2^ > σ(*F*^2^) is used only for calculating *R*-factors(gt) etc. and is not relevant to the choice of reflections for refinement. *R*-factors based on *F*^2^ are statistically about twice as large as those based on *F*, and *R*- factors based on ALL data will be even larger.

### Fractional atomic coordinates and isotropic or equivalent isotropic displacement parameters (Å^2^)

**Table:**

	*x*	*y*	*z*	*U*_iso_*/*U*_eq_	
Zn	0.42078 (3)	0.23364 (2)	0.254511 (13)	0.03328 (8)	
N1	0.21801 (19)	0.10323 (18)	0.17515 (9)	0.0351 (4)	
C1	0.1234 (2)	0.1508 (2)	0.14209 (11)	0.0353 (5)	
H1A	0.1526	0.2422	0.1492	0.042*	
C2	−0.0162 (2)	0.0710 (2)	0.09761 (12)	0.0355 (5)	
H2A	−0.0790	0.1081	0.0755	0.043*	
C3	−0.0604 (2)	−0.0650 (2)	0.08669 (11)	0.0336 (5)	
C4	0.0375 (3)	−0.1155 (2)	0.12028 (14)	0.0457 (6)	
H4A	0.0115	−0.2064	0.1141	0.055*	
C5	0.1747 (3)	−0.0276 (2)	0.16317 (14)	0.0463 (6)	
H5A	0.2407	−0.0617	0.1850	0.056*	
S1	−0.24164 (6)	−0.16230 (6)	0.03032 (3)	0.03988 (14)	
S2	−0.24204 (6)	−0.34128 (6)	0.00746 (3)	0.04027 (14)	
C6	−0.3406 (2)	−0.4474 (2)	0.07251 (11)	0.0344 (5)	
C7	−0.3961 (3)	−0.4103 (2)	0.12877 (13)	0.0476 (6)	
H7A	−0.3824	−0.3226	0.1357	0.057*	
C8	−0.4725 (3)	−0.5053 (2)	0.17481 (13)	0.0466 (6)	
H8A	−0.5116	−0.4795	0.2118	0.056*	
N2	−0.4932 (2)	−0.63104 (18)	0.16917 (9)	0.0339 (4)	
C9	−0.4425 (2)	−0.6675 (2)	0.11302 (12)	0.0377 (5)	
H9A	−0.4595	−0.7563	0.1069	0.045*	
C10	−0.3665 (2)	−0.5801 (2)	0.06409 (12)	0.0377 (5)	
H10A	−0.3330	−0.6094	0.0260	0.045*	
N3	0.61670 (19)	0.37405 (18)	0.33457 (10)	0.0355 (4)	
C11	0.5949 (2)	0.4081 (2)	0.40060 (12)	0.0358 (5)	
H11A	0.4977	0.3640	0.4128	0.043*	
C12	0.7090 (2)	0.5050 (2)	0.45119 (12)	0.0359 (5)	
H12A	0.6889	0.5262	0.4962	0.043*	
C13	0.8547 (2)	0.5704 (2)	0.43370 (11)	0.0335 (5)	
C14	0.8780 (2)	0.5378 (2)	0.36502 (12)	0.0398 (5)	
H14A	0.9735	0.5818	0.3509	0.048*	
C15	0.7571 (2)	0.4390 (2)	0.31818 (12)	0.0415 (5)	
H15A	0.7740	0.4163	0.2726	0.050*	
S3	0.99682 (6)	0.69153 (6)	0.50235 (3)	0.04202 (15)	
S4	1.20007 (6)	0.72797 (6)	0.47202 (3)	0.04384 (15)	
C16	1.2462 (2)	0.8675 (2)	0.41869 (11)	0.0339 (5)	
C17	1.1450 (2)	0.9135 (2)	0.38416 (12)	0.0379 (5)	
H17A	1.0420	0.8714	0.3883	0.046*	
C18	1.2001 (2)	1.0225 (2)	0.34368 (13)	0.0402 (5)	
H18A	1.1317	1.0540	0.3214	0.048*	
N4	1.34608 (19)	1.08651 (18)	0.33422 (10)	0.0357 (4)	
C19	1.4430 (2)	1.0421 (2)	0.36901 (12)	0.0393 (5)	
H19A	1.5457	1.0869	0.3644	0.047*	
C20	1.3994 (2)	0.9344 (2)	0.41103 (12)	0.0381 (5)	
H20A	1.4707	0.9066	0.4339	0.046*	
O1	0.29666 (17)	0.33877 (16)	0.27800 (8)	0.0414 (4)	
O2	0.2410 (2)	0.3097 (2)	0.39220 (9)	0.0544 (5)	
C21	0.2319 (3)	0.3518 (2)	0.33031 (13)	0.0389 (5)	
C22	0.1335 (4)	0.4250 (4)	0.31603 (16)	0.0678 (9)	
H22A	0.0295	0.3630	0.3204	0.081*	
H22B	0.1692	0.4966	0.3551	0.081*	
C23	0.1282 (3)	0.4843 (3)	0.24333 (15)	0.0494 (6)	
C24	0.2307 (3)	0.6123 (3)	0.23429 (19)	0.0666 (8)	
H24A	0.3012	0.6656	0.2750	0.080*	
C25	0.2315 (5)	0.6642 (4)	0.1657 (2)	0.0866 (11)	
H25A	0.3032	0.7509	0.1605	0.104*	
C26	0.1280 (6)	0.5887 (6)	0.1063 (2)	0.0942 (13)	
H26A	0.1294	0.6230	0.0601	0.113*	
C27	0.0224 (5)	0.4634 (5)	0.1139 (2)	0.0892 (12)	
H27A	−0.0500	0.4121	0.0732	0.107*	
C28	0.0224 (4)	0.4120 (3)	0.18197 (19)	0.0687 (8)	
H28A	−0.0513	0.3259	0.1866	0.082*	
O3	0.57013 (19)	0.14005 (17)	0.23618 (8)	0.0453 (4)	
O4	0.5095 (3)	0.0218 (2)	0.12864 (11)	0.0731 (6)	
C29	0.5861 (3)	0.0632 (2)	0.19184 (13)	0.0426 (5)	
C30	0.7158 (4)	0.0237 (3)	0.21550 (17)	0.0652 (8)	
H30A	0.7119	−0.0377	0.1765	0.078*	
H30B	0.8123	0.1035	0.2188	0.078*	
C31	0.7172 (3)	−0.0404 (3)	0.28744 (15)	0.0469 (6)	
C32	0.6281 (3)	−0.1760 (3)	0.28977 (17)	0.0618 (7)	
H32A	0.5654	−0.2267	0.2466	0.074*	
C33	0.6295 (4)	−0.2379 (3)	0.35429 (19)	0.0706 (8)	
H33A	0.5680	−0.3294	0.3543	0.085*	
C34	0.7211 (4)	−0.1654 (3)	0.41871 (18)	0.0663 (8)	
H34A	0.7226	−0.2070	0.4625	0.080*	
C35	0.8096 (4)	−0.0319 (3)	0.41752 (18)	0.0669 (8)	
H35A	0.8717	0.0182	0.4610	0.080*	
C36	0.8088 (3)	0.0305 (3)	0.35277 (17)	0.0605 (7)	
H36A	0.8710	0.1219	0.3532	0.073*	
O5	0.3132 (3)	−0.1386 (2)	−0.00858 (12)	0.0856 (7)	
H5C	0.3720	−0.0885	−0.0334	0.128*	
H5D	0.3676	−0.1044	0.0305	0.128*	

### Atomic displacement parameters (Å^2^)

**Table:**

	*U*^11^	*U*^22^	*U*^33^	*U*^12^	*U*^13^	*U*^23^
Zn	0.03625 (14)	0.03132 (15)	0.02738 (13)	0.01058 (10)	0.00539 (10)	0.00328 (10)
N1	0.0369 (9)	0.0334 (10)	0.0314 (9)	0.0130 (8)	0.0037 (7)	0.0012 (8)
C1	0.0399 (11)	0.0305 (12)	0.0318 (11)	0.0115 (9)	0.0082 (9)	0.0051 (9)
C2	0.0368 (11)	0.0356 (13)	0.0333 (11)	0.0150 (9)	0.0071 (9)	0.0074 (9)
C3	0.0330 (10)	0.0357 (12)	0.0280 (10)	0.0101 (9)	0.0095 (8)	0.0065 (9)
C4	0.0469 (13)	0.0306 (13)	0.0532 (15)	0.0148 (10)	−0.0018 (11)	−0.0016 (11)
C5	0.0451 (13)	0.0415 (15)	0.0502 (15)	0.0213 (11)	−0.0035 (11)	−0.0019 (11)
S1	0.0346 (3)	0.0371 (3)	0.0383 (3)	0.0079 (2)	0.0035 (2)	0.0064 (2)
S2	0.0414 (3)	0.0367 (3)	0.0326 (3)	0.0064 (2)	0.0119 (2)	0.0028 (2)
C6	0.0305 (10)	0.0361 (12)	0.0299 (11)	0.0087 (8)	0.0052 (8)	0.0044 (9)
C7	0.0703 (16)	0.0278 (12)	0.0451 (14)	0.0171 (11)	0.0255 (12)	0.0053 (10)
C8	0.0664 (16)	0.0365 (14)	0.0415 (13)	0.0211 (11)	0.0271 (12)	0.0061 (11)
N2	0.0400 (9)	0.0293 (10)	0.0302 (9)	0.0128 (7)	0.0085 (7)	0.0043 (7)
C9	0.0453 (12)	0.0363 (13)	0.0319 (11)	0.0186 (10)	0.0064 (9)	0.0033 (9)
C10	0.0437 (12)	0.0403 (13)	0.0323 (11)	0.0198 (10)	0.0117 (9)	0.0034 (10)
N3	0.0365 (9)	0.0358 (11)	0.0327 (10)	0.0151 (8)	0.0049 (7)	0.0008 (8)
C11	0.0371 (11)	0.0359 (12)	0.0329 (11)	0.0138 (9)	0.0086 (9)	0.0051 (9)
C12	0.0457 (12)	0.0333 (12)	0.0289 (11)	0.0169 (9)	0.0088 (9)	0.0050 (9)
C13	0.0400 (11)	0.0277 (11)	0.0313 (11)	0.0148 (9)	0.0026 (9)	0.0064 (9)
C14	0.0337 (11)	0.0425 (14)	0.0389 (12)	0.0129 (9)	0.0066 (9)	0.0007 (10)
C15	0.0382 (12)	0.0495 (15)	0.0356 (12)	0.0177 (10)	0.0084 (9)	−0.0034 (10)
S3	0.0443 (3)	0.0374 (3)	0.0313 (3)	0.0071 (2)	0.0037 (2)	0.0024 (2)
S4	0.0408 (3)	0.0383 (3)	0.0477 (3)	0.0154 (2)	0.0009 (2)	0.0144 (3)
C16	0.0394 (11)	0.0294 (12)	0.0305 (11)	0.0140 (9)	0.0036 (9)	0.0038 (9)
C17	0.0320 (11)	0.0377 (13)	0.0421 (13)	0.0129 (9)	0.0085 (9)	0.0107 (10)
C18	0.0376 (11)	0.0454 (14)	0.0421 (13)	0.0218 (10)	0.0088 (10)	0.0140 (11)
N4	0.0365 (9)	0.0342 (10)	0.0350 (10)	0.0136 (8)	0.0084 (8)	0.0078 (8)
C19	0.0312 (10)	0.0495 (15)	0.0341 (12)	0.0149 (10)	0.0062 (9)	0.0055 (10)
C20	0.0348 (11)	0.0452 (14)	0.0362 (12)	0.0203 (10)	0.0035 (9)	0.0070 (10)
O1	0.0494 (9)	0.0504 (10)	0.0324 (8)	0.0286 (8)	0.0101 (7)	0.0016 (7)
O2	0.0647 (11)	0.0735 (13)	0.0402 (10)	0.0405 (10)	0.0204 (8)	0.0146 (9)
C21	0.0413 (12)	0.0386 (13)	0.0377 (12)	0.0171 (10)	0.0113 (10)	0.0019 (10)
C22	0.092 (2)	0.087 (2)	0.0610 (18)	0.0650 (19)	0.0367 (17)	0.0286 (16)
C23	0.0567 (15)	0.0557 (17)	0.0517 (15)	0.0366 (13)	0.0176 (12)	0.0113 (13)
C24	0.0659 (18)	0.060 (2)	0.073 (2)	0.0278 (15)	0.0109 (15)	0.0058 (16)
C25	0.103 (3)	0.067 (2)	0.111 (3)	0.048 (2)	0.043 (3)	0.041 (2)
C26	0.134 (4)	0.133 (4)	0.068 (2)	0.102 (3)	0.028 (3)	0.033 (3)
C27	0.093 (3)	0.123 (4)	0.065 (2)	0.068 (3)	−0.0098 (19)	−0.012 (2)
C28	0.0663 (18)	0.060 (2)	0.081 (2)	0.0295 (15)	0.0121 (16)	−0.0011 (17)
O3	0.0613 (10)	0.0496 (10)	0.0361 (9)	0.0325 (8)	0.0156 (8)	0.0069 (8)
O4	0.0900 (15)	0.0695 (15)	0.0499 (12)	0.0298 (11)	0.0025 (10)	−0.0152 (10)
C29	0.0546 (14)	0.0338 (13)	0.0398 (13)	0.0164 (10)	0.0189 (11)	0.0083 (10)
C30	0.080 (2)	0.072 (2)	0.0706 (19)	0.0493 (17)	0.0411 (16)	0.0242 (16)
C31	0.0523 (14)	0.0432 (15)	0.0565 (15)	0.0286 (11)	0.0183 (12)	0.0071 (12)
C32	0.0708 (18)	0.0469 (17)	0.0577 (18)	0.0215 (14)	−0.0027 (14)	−0.0036 (14)
C33	0.086 (2)	0.0390 (17)	0.078 (2)	0.0230 (14)	0.0032 (17)	0.0111 (15)
C34	0.081 (2)	0.075 (2)	0.0628 (19)	0.0527 (18)	0.0094 (16)	0.0146 (17)
C35	0.0722 (19)	0.070 (2)	0.0612 (19)	0.0390 (17)	−0.0052 (15)	−0.0109 (16)
C36	0.0598 (16)	0.0419 (16)	0.075 (2)	0.0192 (12)	0.0097 (14)	−0.0055 (15)
O5	0.0779 (14)	0.0842 (17)	0.0699 (15)	0.0144 (12)	0.0105 (11)	−0.0102 (12)

### Geometric parameters (Å, °)

**Table:**

Zn—O1	2.1105 (15)	C17—C18	1.371 (3)
Zn—N4^i^	2.160 (2)	C17—H17A	0.9300
Zn—N1	2.180 (2)	C18—N4	1.338 (3)
Zn—N2^ii^	2.1808 (19)	C18—H18A	0.9300
Zn—N3	2.186 (2)	N4—C19	1.338 (3)
Zn—O3	2.2019 (16)	N4—Zn^ii^	2.160 (2)
N1—C5	1.332 (3)	C19—C20	1.371 (3)
N1—C1	1.333 (3)	C19—H19A	0.9300
C1—C2	1.386 (3)	C20—H20A	0.9300
C1—H1A	0.9300	O1—C21	1.270 (2)
C2—C3	1.383 (3)	O2—C21	1.235 (3)
C2—H2A	0.9300	C21—C22	1.512 (4)
C3—C4	1.385 (3)	C22—C23	1.494 (4)
C3—S1	1.776 (2)	C22—H22A	0.9700
C4—C5	1.381 (3)	C22—H22B	0.9700
C4—H4A	0.9300	C23—C24	1.369 (4)
C5—H5A	0.9300	C23—C28	1.375 (4)
S1—S2	2.0311 (10)	C24—C25	1.387 (5)
S2—C6	1.770 (2)	C24—H24A	0.9300
C6—C7	1.377 (3)	C25—C26	1.351 (6)
C6—C10	1.387 (3)	C25—H25A	0.9300
C7—C8	1.381 (3)	C26—C27	1.351 (6)
C7—H7A	0.9300	C26—H26A	0.9300
C8—N2	1.324 (3)	C27—C28	1.376 (5)
C8—H8A	0.9300	C27—H27A	0.9300
N2—C9	1.343 (3)	C28—H28A	0.9300
N2—Zn^i^	2.1808 (19)	O3—C29	1.251 (3)
C9—C10	1.376 (3)	O4—C29	1.242 (3)
C9—H9A	0.9300	C29—C30	1.528 (4)
C10—H10A	0.9300	C30—C31	1.504 (4)
N3—C15	1.335 (3)	C30—H30A	0.9700
N3—C11	1.341 (3)	C30—H30B	0.9700
C11—C12	1.375 (3)	C31—C36	1.379 (4)
C11—H11A	0.9300	C31—C32	1.380 (4)
C12—C13	1.387 (3)	C32—C33	1.373 (4)
C12—H12A	0.9300	C32—H32A	0.9300
C13—C14	1.387 (3)	C33—C34	1.372 (4)
C13—S3	1.773 (2)	C33—H33A	0.9300
C14—C15	1.376 (3)	C34—C35	1.357 (4)
C14—H14A	0.9300	C34—H34A	0.9300
C15—H15A	0.9300	C35—C36	1.379 (4)
S3—S4	2.0294 (10)	C35—H35A	0.9300
S4—C16	1.764 (2)	C36—H36A	0.9300
C16—C17	1.382 (3)	O5—H5C	0.8098
C16—C20	1.392 (3)	O5—H5D	0.8029
			
O1—Zn—N4^i^	97.17 (7)	C17—C16—S4	126.14 (17)
O1—Zn—N1	87.04 (7)	C20—C16—S4	115.38 (17)
N4^i^—Zn—N1	88.63 (7)	C18—C17—C16	118.5 (2)
O1—Zn—N2^ii^	88.99 (7)	C18—C17—H17A	120.7
N4^i^—Zn—N2^ii^	173.74 (7)	C16—C17—H17A	120.7
N1—Zn—N2^ii^	90.57 (7)	N4—C18—C17	124.0 (2)
O1—Zn—N3	88.99 (7)	N4—C18—H18A	118.0
N4^i^—Zn—N3	92.71 (7)	C17—C18—H18A	118.0
N1—Zn—N3	175.94 (7)	C19—N4—C18	116.7 (2)
N2^ii^—Zn—N3	88.51 (7)	C19—N4—Zn^ii^	119.65 (14)
O1—Zn—O3	174.45 (6)	C18—N4—Zn^ii^	122.96 (15)
N4^i^—Zn—O3	84.12 (7)	N4—C19—C20	123.7 (2)
N1—Zn—O3	98.39 (7)	N4—C19—H19A	118.2
N2^ii^—Zn—O3	89.85 (7)	C20—C19—H19A	118.2
N3—Zn—O3	85.56 (7)	C19—C20—C16	118.6 (2)
C5—N1—C1	117.18 (19)	C19—C20—H20A	120.7
C5—N1—Zn	122.60 (15)	C16—C20—H20A	120.7
C1—N1—Zn	119.88 (15)	C21—O1—Zn	137.60 (16)
N1—C1—C2	123.3 (2)	O2—C21—O1	126.2 (2)
N1—C1—H1A	118.4	O2—C21—C22	116.3 (2)
C2—C1—H1A	118.4	O1—C21—C22	117.5 (2)
C3—C2—C1	118.7 (2)	C23—C22—C21	117.5 (2)
C3—C2—H2A	120.6	C23—C22—H22A	107.9
C1—C2—H2A	120.6	C21—C22—H22A	107.9
C2—C3—C4	118.5 (2)	C23—C22—H22B	107.9
C2—C3—S1	116.61 (17)	C21—C22—H22B	107.9
C4—C3—S1	124.88 (18)	H22A—C22—H22B	107.2
C5—C4—C3	118.4 (2)	C24—C23—C28	116.9 (3)
C5—C4—H4A	120.8	C24—C23—C22	121.5 (3)
C3—C4—H4A	120.8	C28—C23—C22	121.6 (3)
N1—C5—C4	123.9 (2)	C23—C24—C25	121.4 (3)
N1—C5—H5A	118.1	C23—C24—H24A	119.3
C4—C5—H5A	118.1	C25—C24—H24A	119.3
C3—S1—S2	104.60 (9)	C26—C25—C24	119.9 (4)
C6—S2—S1	105.51 (8)	C26—C25—H25A	120.1
C7—C6—C10	118.0 (2)	C24—C25—H25A	120.1
C7—C6—S2	125.76 (19)	C25—C26—C27	120.2 (4)
C10—C6—S2	116.21 (16)	C25—C26—H26A	119.9
C6—C7—C8	119.0 (2)	C27—C26—H26A	119.9
C6—C7—H7A	120.5	C26—C27—C28	119.8 (4)
C8—C7—H7A	120.5	C26—C27—H27A	120.1
N2—C8—C7	123.7 (2)	C28—C27—H27A	120.1
N2—C8—H8A	118.2	C23—C28—C27	121.9 (3)
C7—C8—H8A	118.2	C23—C28—H28A	119.1
C8—N2—C9	117.00 (19)	C27—C28—H28A	119.1
C8—N2—Zn^i^	121.68 (14)	C29—O3—Zn	143.09 (17)
C9—N2—Zn^i^	120.90 (15)	O4—C29—O3	125.0 (2)
N2—C9—C10	123.3 (2)	O4—C29—C30	117.4 (2)
N2—C9—H9A	118.4	O3—C29—C30	117.5 (2)
C10—C9—H9A	118.4	C31—C30—C29	117.2 (2)
C9—C10—C6	118.9 (2)	C31—C30—H30A	108.0
C9—C10—H10A	120.5	C29—C30—H30A	108.0
C6—C10—H10A	120.5	C31—C30—H30B	108.0
C15—N3—C11	117.28 (19)	C29—C30—H30B	108.0
C15—N3—Zn	122.71 (14)	H30A—C30—H30B	107.3
C11—N3—Zn	119.66 (14)	C36—C31—C32	117.1 (3)
N3—C11—C12	123.3 (2)	C36—C31—C30	122.5 (3)
N3—C11—H11A	118.4	C32—C31—C30	120.3 (3)
C12—C11—H11A	118.4	C33—C32—C31	121.7 (3)
C11—C12—C13	118.72 (19)	C33—C32—H32A	119.2
C11—C12—H12A	120.6	C31—C32—H32A	119.2
C13—C12—H12A	120.6	C34—C33—C32	120.3 (3)
C14—C13—C12	118.5 (2)	C34—C33—H33A	119.9
C14—C13—S3	125.30 (17)	C32—C33—H33A	119.9
C12—C13—S3	116.18 (16)	C35—C34—C33	118.9 (3)
C15—C14—C13	118.6 (2)	C35—C34—H34A	120.6
C15—C14—H14A	120.7	C33—C34—H34A	120.6
C13—C14—H14A	120.7	C34—C35—C36	120.9 (3)
N3—C15—C14	123.5 (2)	C34—C35—H35A	119.5
N3—C15—H15A	118.2	C36—C35—H35A	119.5
C14—C15—H15A	118.2	C31—C36—C35	121.1 (3)
C13—S3—S4	105.43 (8)	C31—C36—H36A	119.5
C16—S4—S3	106.12 (8)	C35—C36—H36A	119.5
C17—C16—C20	118.5 (2)	H5C—O5—H5D	95.1

Symmetry codes: (i) *x*−1, *y*−1, *z*; (ii) *x*+1, *y*+1, *z*.

### Hydrogen-bond geometry (Å, °)

**Table:**

*D*—H···*A*	*D*—H	H···*A*	*D*···*A*	*D*—H···*A*
O5—H5c···O4^iii^	0.81	2.20	2.970 (3)	158
O5—H5d···O4	0.80	2.15	2.945 (3)	169

Symmetry codes: (iii) −*x*+1, −*y*, −*z*.
